# Circumstances Preceding Firearm Suicide Deaths in a Large Sample of Hispanic/Latino Adults: Findings From a Content Analysis

**DOI:** 10.1016/j.focus.2026.100531

**Published:** 2026-07-15

**Authors:** Alexys M. Abraham, Elise V. Bailey, Aryanna Sanger, Diana E. Naranjo, Fernando A. Wilson, Evan V. Goldstein

**Affiliations:** 1 Department of Population Health Sciences, Spencer Fox Eccles School of Medicine, University of Utah, Salt Lake City, Utah; 2 Department of Internal Medicine, Spencer Fox Eccles School of Medicine, University of Utah, Salt Lake City, Utah; 3 Informatics, Decision-Enhancement and Analytic Sciences (IDEAS) Center, VA Salt Lake City Health Care System, Salt Lake City, Utah; 4 Department of Economics, University of Utah, Salt Lake City, Utah; 5 Matheson Center for Health Care Studies, University of Utah, Salt Lake City, Utah

**Keywords:** Firearms, weapons, Hispanic, Latino, suicide

## Abstract

**Introduction:**

Rates of suicide deaths involving firearms have been increasing among Hispanic/Latino adults in the U.S. The high lethality of firearm suicide attempts and critical gaps in characterizing firearm suicide deaths among Hispanic/Latino adults may pose challenges to suicide prevention. However, there is little systematic knowledge about the circumstances that manifest among Hispanic/Latino suicide decedents who die by firearms or about how those circumstances may differ between female and male Hispanic/Latino firearm suicide decedents. This study aimed to descriptively characterize recurring circumstances preceding firearm suicide deaths among a large sample of Hispanic/Latino adults in the U.S. and to examine sex differences in the occurrence of these circumstances.

**Methods:**

This study analyzed deidentified data from the National Violent Death Reporting System. The analysis included a large sample of 1,170 Hispanic/Latino adult firearm suicide decedents who died from 2013 to 2021 in the U.S. Given the limited etiologic characterization of firearm suicide among Hispanic/Latino adults, a qualitative content analysis with quantification was conducted to systematically review and code recurring circumstances experienced prior to death using free-text coroner/medical examiner and law enforcement narratives abstracted by the National Violent Death Reporting System. This study grounded circumstance definitions inductively, directly in the language and contextual descriptions present in the narrative data.

**Results:**

Six major categories emerged to organize the recurring circumstances preceding firearm suicide deaths among the individuals included in the study sample: behavioral health, physical health, relationship dynamics, socioeconomic instability, scene of death, and firearm access and storage. Behavioral health circumstances were commonly noted among the decedents in the sample, including known mental health conditions and depressed or melancholy mood. Relationship and socioeconomic stressors were noted for a meaningful portion of the decedents in the sample, including romantic relationship challenges, interpersonal arguments, and legal issues. Notable sex differences were observed; female decedents in the sample were more frequently documented as having prior suicide attempts and accessing another person’s firearm.

**Conclusions:**

Although descriptive, these findings provide population-specific context on the circumstances preceding firearm suicide among Hispanic/Latino adults, a group historically underrepresented in the firearm suicide literature. Several findings extend existing knowledge to a population for whom such evidence remains limited. Many identified circumstances are consistent with prior research on suicide broadly, underscoring their relevance within this population. In addition, this study’s findings align with existing National Violent Death Reporting System variables, offering insight into the quality and utility of National Violent Death Reporting System data. This study provides a foundation for subsequent hypothesis-driven research on circumstances and risk factors preceding firearm suicide among Hispanic/Latino adults.

## HIGHLIGHTS

• This study analyzed National Violent Death Reporting System narrative data on Hispanic/Latino adult firearm suicides.
• Circumstances before death spanned health, social, economic, and firearm domains.
• The commonality of several circumstances documented in the data varied by sex.
• Findings add population-specific context consistent with previous research.
• Findings aligned with structured National Violent Death Reporting System variables, supporting data quality and utility.

## INTRODUCTION

Suicide rates increased by 40.8% among U.S. Hispanic/Latino adults (aged ≥18 years) from 2013 to 2021 (i.e., from 7.41 to 10.43 per 100,000 people), compared with an 8.2% rise in non-Hispanic/Latino adult suicide rates over the same period.^1^ Although many people who attempt suicide survive,^2^ attempts involving firearms are more likely to be fatal than other methods (e.g., poison). Previous studies have demonstrated that firearms represent the most lethal and irreversible suicide method, with case-fatality rates estimated at 80%–90%.^3^^,^^4^ Although firearms alone do not cause individuals to become suicidal, numerous studies have demonstrated significantly higher odds of suicide death among persons with access to firearms in their homes than among those without access to firearms.^5^ Firearm industry survey data suggest that firearm purchasing has increased significantly among Hispanic/Latino persons in recent years,^6^ a trend that may elevate population-level risk for firearm suicide, given the association between firearm access and suicide mortality. Notably, suicide rates involving firearms among Hispanic/Latino adults increased by 58.9% from 2013 to 2021 (i.e., from 2.75 to 4.37 per 100,000 people), compared with a 30.0% increase in nonfirearm suicide deaths among Hispanic/Latino adults over the same period.^1^ Firearm suicide deaths accounted for 327,247 years of potential life lost among Hispanic/Latino adults across 2013–2021.^1^

The high lethality of firearm suicide attempts poses significant prevention challenges. Previous studies have demonstrated that many persons who have made serious suicide attempts thought about suicide for as little as 5–10 minutes before they acted,^7^ so safe firearm storage (or voluntary firearm removal) may make the difference between life and death among people at risk. Previous research has also suggested that when one method of suicide is not accessible, individuals rarely substitute different suicide methods.^8^ Even if other suicide methods are substituted, the likelihood of death from the alternative methods may be reduced, given the lethality of firearm-involved attempts. This underscores the importance of identifying the circumstances that characterize firearm suicide deaths among Hispanic/Latino adults. Although previous studies have identified factors associated with suicidal ideation in Hispanic/Latino communities—including mental ill health, acculturative stress, economic instability, poverty, and discrimination^9^, ^10^, ^11^, ^12^, ^13^ —this evidence derives primarily from research on suicide broadly. Little systematic knowledge exists regarding the specific circumstances preceding firearm suicide deaths among Hispanic/Latino individuals.^14^ Moreover, recent studies have demonstrated that the circumstances experienced by firearm suicide decedents may vary by race, ethnicity, and sex,^14^^,^^15^ which may have implications for understanding the etiology of firearm suicide death in Hispanic/Latino communities and for developing population-responsive prevention strategies. Notably, these knowledge gaps have emerged in the context of the pandemic-era erosion of the longstanding Latino health advantage in the U.S.—particularly among middle-aged Hispanic/Latino adults—potentially reflecting broader shifts in social, economic, and health-related stressors that may also shape suicide risk.^16^

Sex differences may further shape the epidemiology and contextual circumstances of firearm suicide among Hispanic/Latino adults. Previous research has indicated that males and females differ in suicide mortality, method selection, demographic characteristics, mental health burden, and exposure to social and economic stressors, pointing to potentially distinct etiologic pathways to death by firearm suicide.^15^^,^^17^, ^18^, ^19^ Moreover, recent evidence suggests that many new firearm owners in the U.S. from 2019 to 2021 were female.^20^ Recent research has also demonstrated that secondary exposure to household firearms is associated with higher suicide rates among women who do not personally own handguns, highlighting the evolving nature of the risk of suicide involving firearms among women.^21^ Among female Hispanic/Latina adults, suicide rates involving firearms increased by 53.0% from 2013 to 2021 (i.e., from 0.66 to 1.01 per 100,000 people).^1^ However, only a small number of studies have examined firearm suicide among Hispanic/Latino adults in recent years,^9^^,^^14^^,^^22^ and the systematic characterization of the circumstances preceding firearm suicide, among Hispanic/Latina females is especially limited.

To address these knowledge gaps, this study conducted a qualitative content analysis of National Violent Death Reporting System (NVDRS) narrative data using an inductive coding process followed by deductive code application for quantification. The objective was to descriptively characterize recurring circumstances preceding firearm suicide deaths among a large sample of Hispanic/Latino adults in the U.S. and to examine sex differences in the occurrence of these circumstances. Consistent with this objective, this study was not designed to compare firearm and nonfirearm suicide deaths but rather to characterize circumstances within the firearm suicide decedent population. Accordingly, the analysis was designed to support etiologic understanding in a large sample of decedents and to identify circumstances for use in subsequent hypothesis-driven analyses, including those that may extend beyond variables currently captured by NVDRS. In addition, this study provides a methodologic contribution by showing how qualitative content analysis of NVDRS narrative data can add contextual detail and nuance to structured variables for Hispanic/Latino populations. The findings also demonstrate the utility of NVDRS narrative data, particularly through the observed consistency between narrative-derived circumstances and existing structured variables.

## METHODS

### Study Sample

This study received deidentified data from the NVDRS Restricted Access Database^23^ on Hispanic/Latino adult firearm suicide deaths from 2013 to 2021 through an approval process managed by the Centers for Disease Control and Prevention. The NVDRS program incorporates data from vital records and investigative reports from coroners/medical examiners (CMEs) and law enforcement (LE) and compiles comprehensive incident-level data about violent deaths in the U.S. This study examined the free-text CME/LE narratives summarized by the NVDRS program from interviews of persons close to each decedent, including family members, spouses, and next of kin, which provide relevant information about the circumstances occurring prior to each death.

The analytic sample was selected using the following inclusion criteria. First, individuals who died by suicide involving firearms were included. These individuals were identified using the NVDRS program’s process for determining suicide—which standardizes manner of death designations from death certificates and CME investigations across many different jurisdictions—and the NVDRS program’s standardized information on the weapon resulting in each death (i.e., firearm).^23^ Second, only individuals with Hispanic ethnicity were included. The NVDRS program follows the HHS standards for ethnicity categorization. Specifically, decedents with Mexican, Puerto Rican, Cuban, Central or South American, or other Spanish culture or origin are coded as Hispanic/Latino, regardless of race. Third, decedents aged ≥18 years at death were included. Fourth, to conduct the content analysis, decedents with available CME or LE narrative data were included,^24^^,^^25^ given that identification and characterization of the circumstances preceding death were not feasible in the absence of qualitative data.

As described by Krippendorff (2004)^26^ and similar to other studies using NVDRS data,^27^, ^28^, ^29^ the analytic sample was selected using a probability sampling approach, permitting the study team to analyze a large corpus of qualitative data within the resource and time constraints of the grant funding this project. The study team selected a 20% simple random sample without replacement from all Hispanic/Latino adult firearm suicide decedents with nonmissing CME/LE information in the NVDRS from 2013 to 2021. Of note, about 4.4% of Hispanic/Latino adult firearm suicide decedents in the NVDRS during this period had missing CME/LE narrative data before sample selection. Although statistical generalization was not the objective, before analysis, the study team compared demographic, temporal, and geographic characteristics between sampled and nonsampled individuals, including those with missing CME/LE narrative data, using absolute standardized differences (ASDs). ASDs quantify the magnitude of differences in distributions independent of sample size, with values <0.10 conventionally interpreted as indicating negligible imbalance.^30^ Ultimately, the analytic sample appeared to be representative of all Hispanic/Latino adult firearm suicide decedents in the available NVDRS data from 2013 to 2021, including those with missing CME/LE narrative data, on measured characteristics (all ASDs <0.10) (Table A.1, available online). Consistent with the study’s objective and qualitative content analytic approach, analyses were restricted to firearm suicide decedents rather than being designed to compare across mechanisms of death.

The final analytic sample included 1,170 decedents from 46 states, including the District of Columbia and Puerto Rico (Table A.2, available online, provides a complete list of states and contributing years). This study was deemed nonhuman subjects research by the University of Utah IRB (IRB_00181189) because it used deidentified surveillance data on deceased persons only.

### Analysis

Given the limited etiologic characterization of firearm suicide among Hispanic/Latino adults, this study employed a qualitative approach to systematically review and code recurring circumstances described in NVDRS CME/LE narrative data. Rather than restricting the analysis to predefined structured circumstance variables in the NVDRS, this study allowed circumstances to emerge directly from the narrative text to capture contextual nuance and population-specific framing. Although preexisting NVDRS variables capture whether selected circumstances were present at the time of death, such variables reflect the application of a predefined surveillance taxonomy. The narrative texts may contain additional detail regarding how stressors are described, contextualized, and temporally framed. Notably, prior research has demonstrated that NVDRS narrative data can vary systematically across racial/ethnic groups in both language and informational content. Rahman et al.^31^ (2022) found that phrases and words most predictive of suicide can differ between Black and White decedents and that, in some scenarios, Black decedents’ narratives were significantly shorter than White decedents’ narratives in the number of characters, words, and lexical diversity. In addition, Mezuk and colleagues^32^ (2021) documented racial/ethnic differences in the presence, length, and detail of NVDRS narrative text. Therefore, consistent with the research team’s positionality^33^^,^^34^ (Appendix, available online), this study sought to ground all circumstance definitions directly in the language and contextual descriptions present in the narrative data for Hispanic/Latino decedents rather than limiting definitions solely to predefined NVDRS variables.

Specifically, this study conducted qualitative content analysis with quantification^26^^,^^35^^,^^36^ to identify, describe, and enumerate the recurring circumstances that the decedents experienced prior to death, as summarized through the CME/LE narratives. The CME/LE narrative was the unit of analysis. This process involved 3 systematic phases. In the first phase, the study team inductively identified and defined recurring circumstances from a random subsample of 100 cases and developed a standardized first-cycle codebook.^37^ In the second phase, the existing codes were deductively applied to all cases in each analytic sample, with ongoing team review to ensure consistency; discuss the emergence of any new, salient codes; and make any necessary refinements to the code definitions.^24^^,^^38^ Final reliability checks demonstrated >95% agreement and Gwet’s AC1 values >0.75, indicating substantial interrater reliability and clear, consistent code application across samples. In the third phase, the study team organized the identified circumstances into major categories using focused coding.^37^^,^^39^ This allowed the team to construct analytically meaningful categories while remaining sufficiently flexible to capture the range of circumstances documented in the narratives rather than imposing overly restrictive categories.^37^ Additional details about this process are provided in the Appendix (available online). Of note, the absence of a documented circumstance should not be interpreted as evidence that the circumstance did not occur but rather that it was not known or recorded in the available data.

Finally, the study team summarized the circumstances by sex using proportions. Differences between males and females were quantified as unadjusted risk differences (RDs) (male minus female) with corresponding 95% CIs. To account for multiple comparisons across circumstances, *p*-values from 2-sample tests of proportions were adjusted using the Benjamini–Hochberg false discovery rate (FDR) procedure.^25^ Biological sex was defined according to the NVDRS as per the death certificate. CME/LE data were reviewed, coded, and organized using Dedoose, Version 10.0.35. Statistical analyses were conducted using Stata MP 18.5.

## RESULTS

Table 1 describes the analytic sample. The decedents had a mean age of 38.4 years (range: 18–93 years). Most decedents were male (88.5%). The male and female decedents had mean ages of 38.6 (range: 18–93) and 37.2 (range: 18–76) years, respectively. About 39.9% of the decedents had a high-school diploma or GED, whereas about 8.9% had a bachelor’s, master’s, doctorate, or professional degree. Nearly 3 in 10 (29.7%) decedents were married, in a civil union, or in a domestic partnership at the time of death. About 14.5% of the decedents served in the U.S. Armed Forces.

**Table 1:** Describing the Analytic Sample (N=1,170), 2013–2021

Variable	Mean/frequency	SD/percent
Age in years, mean, SD	38.4	16.4
Sex, *n*, %		
Male	1,036	88.5%
Female	134	11.4%
Education level, *n*, %		
8th grade or less	101	8.8%
9th to 12th grade, no diploma	171	14.8%
High-school graduate or GED completed	460	39.9%
Some college credit but no degree	209	18.1%
Associate's degree	67	5.8%
Bachelor's degree	80	6.9%
Master's, Doctorate, or professional degree	23	2.0%
Unknown	43	3.7%
Marital Status, *n*, %		
Married/civil union/domestic partnership	348	29.7%
Never married	572	48.9%
Widowed	37	3.2%
Divorced	148	12.7%
Married/civil union/domestic partnership but separated	35	3.0%
Single, not otherwise specified	19	1.6%
Unknown	11	0.9%
Military veteran status, *n*, %		
Did not serve	979	83.8%
Served in the U.S. Armed Forces	170	14.5%
Unknown	20	1.7%
Death Year, *n*, %		
2013	39	3.3%
2014	60	5.1%
2015	79	6.8%
2016	94	8.0%
2017	126	10.8%
2018	164	14.0%
2019	145	12.4%
2020	214	18.3%
2021	249	21.3%
Region, *n*, %		
West (CA, OR, WA, AK, HI, NV, AZ, NM, CO, UT, ID, WY, MT)	645	55.1%
Midwest (IL, IN, MI, OH, WI, IA, KS, MN, MO, NE, ND, SD)	172	14.7%
Northeast (CT, ME, MA, RI, NJ, NY, PA)	92	7.9%
South (DC, GA, MD, NC, SC, VA, WV, AL, KY, TN, LA, OK, TX)	228	19.5%
Puerto Rico	33	2.8%

*Note*: This table presents authors' analysis of NVDRS data.

The region is based on U.S. Census Bureau classifications. Puerto Rico is not part of the 4 Census regions and is shown as a separate category.

AK, Alaska; AL, Alabama; AZ, Arizona; CA, California; CO, Colorado; CT, Connecticut; DC, District of Columbia; GA, Georgia; HI, Hawaii; IA, Iowa; ID, Idaho; IL, Illinois; IN, Indiana; KA, Kansas; KY, Kentucky; LA, Los Angeles; MA, Massachusetts; MD, Maryland; ME, Maine; MI, Michigan; MN, Minnesota; MO, Missouri; MT, Montana; NC, North Carolina; ND, North Dakota; NE, Nebraska; NJ, New Jersey; NM, New Mexico; NV, Nevada; NVDRS, National Violent Death Reporting System; NY, New York; OH, Ohio; OK, Oklahoma; OR, Oregon; PA, Pennsylvania; RI, Rhode Island; SC, South Carolina; SD, South Dakota; TN, Tennessee; TX, Texas; UT, Utah; VA, Virginia; WA, Washington; WI, Wisconsin; WV, West Virginia; WY, Wyoming.

Table 2 describes the recurring circumstances that emerged during the content analysis. Six major categories emerged to organize the circumstances: behavioral health, physical health, relationship dynamics, socioeconomic instability, scene of death, and firearm access and storage. Each circumstance coincided with a specific first-cycle code.^37^ In addition to the circumstance descriptions, illustrative text fragments are provided to offer context for how these circumstances were described and documented in the narrative data. Coding definitions and criteria are included in the Appendix (available online).

**Table 2:** Recurring Circumstances Preceding Firearm Suicide Death Among Hispanic/Latino Adults (N=1,170), by Category, 2013–2021

Category/circumstance	Illustrative text fragments	*n*	%
Behavioral health			
Mental health condition known: The decedent was noted to have experienced a mental health/psychiatric condition or disorder preceding death.	“had bipolar disorder,” “had a diagnosis of depression,” “being seen for anxiety”	309	26.4%
Substance/alcohol use history: The decedent was noted to have experienced a known substance use disorder, alcoholism, or patterned history of drug or alcohol use.	“noted history of drug use,” “has a history of alcoholism,” “had a history of chronic alcohol abuse”	202	17.3%
Acute alcohol use: The decedent was noted to have consumed alcohol the day of death. This may have been indicated by a next-of-kin interview, indications of empty alcohol bottles used at the scene of death in combination with supplemental information included in the narrative, such as a toxicology report.	“had been drinking alcohol throughout the day and night,” “alcohol use was observed,” “toxicology was positive for alcohol”	377	32.2%
Acute substance use: The decedent was noted to have used at least 1 recreational/illicit drug the day before death or the day of death.	“spouse had reported their cocaine use the previous night,” “was self-medicating with methamphetamine and heroin,” “tested positive for marijuana”	310	26.5%
History of suicide attempt: The decedent was noted to have made at least 1 additional suicide attempt in the past, different from the attempt resulting in death.	“had a prior suicide attempt,” “attempted to take their own life in their car three years prior,” “attempted to shoot themself one time at their sister’s home”	142	12.1%
Feeling depressed/melancholy: The decedent was noted to have been feeling depressed, dejected, down, or melancholy leading up to death (i.e., not a mental health/psychiatric condition).	“was feeling depressed about it,” “feeling down and lonely,” “seemed depressed about not getting the job”	341	29.2%
Psychiatric/mental health hospitalization: The decedent was noted to have had an inpatient hospitalization, rehabilitation, or residential treatment facility stay for psychiatric/mental health reasons at any time prior to death.	“was hospitalized for a previous suicide attempt,” “was transferred to a psychiatric hospital,” “was hospitalized at a facility for mental illness”	67	5.7%
Physical health			
General medical condition known: The decedent experienced a known physical health/general medical condition preceding death.	“current medical history included hypertension and asthma,” “back issues that caused chronic pain,” “had nerve issues and chronic obstructive pulmonary disorder”	185	15.8%
Physical pain: The decedent was noted to have been experiencing physical pain preceding death.	“could not take the bladder pain,” “complained that they didn’t have any medication for their foot pain,” “had chronic back pain due to a ruptured disk”	72	6.2%
Sleep trouble: The decedent was noted to have experienced trouble sleeping, including conditions such as insomnia and sleep apnea, or sleeping more than they usually did.	“sleeping very little,” “experiencing insomnia,” “couldn’t sleep due to the medications”	33	2.8%
Relationship dynamics			
Argument with romantic partner: The decedent was noted to have had an argument or dispute with their romantic partner (e.g., spouse, boyfriend, girlfriend, partner) during the days prior to death or day of death.	“had started arguing with their boyfriend,” “they had an argument,” “had a fight with their spouse the night before”	208	17.8%
Argument with nonromantic person: The decedent was noted to have an argument or dispute with someone other than a romantic partner (e.g., family member, friend, coworker) during the days prior to death or day of death.	“an argument ensued with their mother,” “during an argument with their son,” “got into a verbal altercation with one of the care providers”	46	3.9%
Romantic relationship challenges: The decedent was noted to have experienced problems in an established romantic relationship.	“had been fighting a lot,” “depressed for the last week due to relationship issues with their wife,” “having marital issues”	279	23.9%
Romantic relationship end/breakup: The decedent was noted to have experienced either the end of a romantic relationship or plans to end a romantic relationship preceding death.	“were in the process of a divorce,” “told them that they wanted to end the relationship,” “had separated and their spouse had kicked them out of the house”	213	18.2%
Known/suspected infidelity: There were noted indications of either known or suspected infidelity, including romantic affairs and cheating.	“found their girlfriend with another person,” “had an affair with their wife’s sister,” “their wife was seeing another person”	69	5.9%
Final expressions of love: The decedent was noted to have expressed their love in combination with an expression of saying goodbye to family members, romantic partners, or friends within the days prior to death or the day of death.	“called their daughter and told her they loved her,” “sent a text to their sister telling her they loved her,” “received a Facebook message saying they loved her late that night”	41	3.5%
Socioeconomic instability			
Job loss/unemployment: The decedent was noted to have experienced either a job loss or unemployment preceding death.	“was recently unemployed,” “lost their job,” “fired from their job one week prior”	83	7.1%
Financial difficulties: The decedent was noted to have experienced a financial setback or financial distress preceding death.	“was recently struggling financially,” “worried about money and losing their home,” “wrote that their financial problems were what led to this decision”	73	6.2%
Legal issues: The decedent was noted to have been experiencing legal troubles or distress preceding death, for example, being arrested, being accused of a crime, being sued, or having immigration-related legal issues.	“had been arrested for DUI,” “dealing with legal problems,” “had come to the United States illegally two years ago and was having immigration issues”	116	9.9%
Housing instability: The decedent was noted to have experienced homelessness or housing instability within the year prior to death.	“was living in their vehicle,” “stressed about losing their home,” “was unable to pay rent and was evicted”	38	3.3%
Scene of death			
Found away from residence: The decedent was noted to have been found away from their primary residence, that is, public places, nature, other people's residences, and businesses.	“found outside [their] residence,” “found in a remote area by a group of campers,” “found at a local farm”	279	23.9%
Found in automobile: The decedent was noted to have been found inside a car or other automobile either at or away from their primary residence.	“was found in their vehicle near an intersection,” “found in their car parked near the home,” “found in their car by a passerby”	143	12.2%
Resuscitation attempt before death: There was a noted attempt to perform resuscitation at the time of death.	“succumbed to their injuries at the emergency department,” “paramedics performed life-saving measures but were unable to save the victim,” “initiated resuscitation without success”	116	9.9%
Firearm access and storage			
Another person’s firearm accessible: The decedent was noted to have accessed (i.e., borrowed or taken) the firearm involved in the fatal suicide attempt from a family member, romantic partner, friend, or nonfamily acquaintance. The firearm was not owned by the decedent.	“the weapon belonged to the victim’s father,” “called their friend a few days before and asked him to bring a firearm to the residence,” “their partner’s gun that he keeps in the nightstand”	75	6.4%
Multiple firearms accessible: The decedent was noted to have multiple firearms accessible at the time of death.	“wife stated victim owned a second firearm,” “there was a 9mm caliber handgun in their left hand and a .22LR caliber handgun in their right hand,” “in the vehicle were two handguns”	54	4.6%
Unsecure firearm storage: The decedent was noted to have not practiced secure firearm storage practices prior to death, including indications of keeping loaded firearms unlocked or stored in an open location in the home or vehicle.	“always keeps a loaded firearm within reach in their bedroom,” “kept their gun in their room in an unsecured backpack,” “kept the handgun between their pillows”	33	2.8%
Firearm acquired legally: The decedent was noted to have acquired the firearm used in the fatal suicide attempt following a legal process for purchasing a firearm.	“was the registered owner of the gun,” “handgun registered to the victim,” “had a valid firearm license”	22	1.9%

*Note*: This table presents the authors' analysis of NVDRS data. The circumstances shown in this table are not mutually exclusive. The absence of a documented circumstance should not be interpreted as evidence that the circumstance did not occur but rather that it was not known or recorded in the available data. Illustrative text fragments from the CME/LE narratives shown in this table were subject to anonymization through a process that omits or reconstructs specific details and uses sex-neutral descriptions of the victims.

CME, coroners/medical examiner; LE, law enforcement; NVDRS, National Violent Death Reporting System.

The *behavioral health* category encompassed mental health and substance-related factors documented in the period preceding death. These circumstances included known psychiatric conditions, substance and alcohol use (acute or historical), prior suicide attempts, symptoms of depressed or melancholy mood, and prior psychiatric or mental health hospitalization. About 1 in 4 (26.4%) firearm suicide decedents in the sample were noted to have experienced a mental health or psychiatric condition or disorder within 1 year of death. Among these decedents, the narrative data most often documented phrases such as "mental health conditions". Other narratives noted specific conditions, including mood disorders (e.g., major depressive disorder, bipolar disorder), anxiety disorders (e.g., generalized anxiety disorder, post-traumatic stress disorder), psychotic disorders (e.g., schizophrenia, delusional disorder), obsessive-compulsive disorder, dissociative disorders, and eating disorders. These were indicated in various ways (Table A.3, available online). Feeling depressed, dejected, down, or melancholy in the period leading up to death was also commonly documented in the sample (29.2% of decedents). These feelings were coded separately from known mental health or psychiatric conditions.

About 1 in 10 (12.1%) decedents were noted to have made at least 1 previous suicide attempt before the attempt resulting in death. About 17.3% of firearm suicide decedents were noted to have experienced a known substance use disorder or a patterned history of drug or alcohol use in the sample. Acute alcohol use and acute recreational or illicit drug use were documented for 32.2% and 26.5% of decedents, respectively. These variables were based on narrative documentation and may not reflect substance quantity or blood alcohol concentration at the time of death. About 5.7% of firearm suicide decedents in the sample were noted to have had an inpatient hospitalization, rehabilitation, or residential treatment facility stay for psychiatric or mental health reasons at any time prior to death.

The *physical health* category captured physical health conditions and somatic symptoms documented in the period preceding death, including known general medical conditions, reported physical pain, and sleep-related problems. Known general medical conditions were noted for 15.8% of the decedents in the sample. Many narratives noted specific chronic conditions among decedents, including heart disease (3.8%), hypertension (2.9%), diabetes mellitus (2.6%), cancer (2.4%), nonback-related chronic pain disorder (1.8%), chronic back pain disorder (1.4%), neurologic disorder (1.4%), hepatic disease (1.2%), and gastrointestinal disease (1.1%). Additional conditions—including asthma, chronic obstructive pulmonary disease, kidney disease, cognitive disease, high cholesterol, chronic vision disease, AIDS, lung disease, and pancreatic disease—were each noted but for fewer than 10 decedents. Many narratives mentioned nonspecific physical health problems. Physical pain and sleep-related difficulties were noted for 6.2% and 2.8% of the decedents, respectively.

The *relationship dynamics* category included circumstances reflecting interpersonal tensions and disruptions in the decedent’s romantic relationships. This encompassed recent arguments, chronic relationship problems, the end or anticipated end of relationships, and indications of known or suspected infidelity. About 1 in 4 (23.9%) firearm suicide decedents were noted to have experienced problems in an established romantic relationship in the months leading up to their death. Recent arguments or disputes with romantic partners (e.g., spouse, boyfriend, girlfriend, or partner) and nonromantic persons were documented for 17.8% and 3.9% of decedents, respectively. Known or suspected infidelity in the relationship (in 5.9% of decedents) and final expressions of love accompanied by a goodbye to family members, romantic partners, or friends (in 3.5% of decedents) were also documented in the sample.

The *socioeconomic instability* category reflected circumstances related to economic hardship and broader socioeconomic stressors noted before death. These experiences included unemployment or job loss, financial strain, housing instability, and legal troubles documented before death. About 7.1% of the decedents in the sample were noted to have experienced either job loss or unemployment preceding death. About 1 in 10 (9.9%) firearm suicide decedents were noted to have been experiencing legal troubles or distress before death (including being arrested, accused of a crime or sexual misconduct, being sued, or experiencing immigration-related legal issues). About 6.2% of the decedents in the sample were noted to have experienced financial difficulties before death.

The *scene of death* category provided information on the location where the decedent was found and the immediate response context, including being discovered away from their residence or in an automobile and whether resuscitation was attempted. About 1 in 4 (23.9%) decedents in the sample were noted to have been found away from their residence. About 12.2% of the decedents in the sample were noted to have been found in an automobile. A resuscitation attempt prior to death was documented for 9.9% of the decedents.

The *firearm access and storage* category captured circumstances noting how the firearm was accessed, the availability of firearms, storage practices, and whether the firearm used in the fatal attempt was legally acquired. About 6.4% of decedents in the sample were noted to have accessed a firearm that they had borrowed, taken, or otherwise obtained from another person. Multiple firearms were noted to be accessible for 4.6% of the decedents, and unsecured firearm storage was noted for 2.8% of the decedents, including indications of loaded firearms that were kept unlocked or stored in open locations in a home or vehicle.

Table 3 describes the circumstances by sex. Several circumstances differed between female and male decedents. Female decedents in the sample were more frequently noted to have known mental health conditions preceding death than males (52.2% vs 23.1%; RD= −29.2, 95% CI= −38.0, −20.3; FDR-adjusted *p*<0.001). Similarly, a history of prior suicide attempt was noted more frequently among females than among males (28.4% vs 10.0%; RD= −18.3, 95% CI= −26.2, −10.5; FDR-adjusted *p*<0.001). Physical pain preceding death was also more frequently documented among female decedents (13.4% vs 5.2%; RD= −8.2, 95% CI= −14.2, −2.3; FDR-adjusted *p*=0.001).

**Table 3:** Recurring Circumstances Preceding Firearm Suicide Death Among Hispanic/Latino Adults, by Category and Sex, 2013–2021

Circumstance	Male (%)	Female (%)	Risk difference (95% CI)	FDR-adjusted *p*-value
Behavioral health				
Mental health condition known: The decedent was noted to have experienced a mental health/psychiatric condition or disorder preceding death.	23.1%	52.2%	−29.2 (−38.0, −20.3)	<0.001
Substance/alcohol use history: The decedent was noted to have experienced a known substance use disorder, alcoholism, or patterned history of drug or alcohol use.	17.1%	18.7%	−1.6 (−8.6, 5.4)	0.858
Acute alcohol use: The decedent was noted to have consumed alcohol the day of death. This may have been indicated by a next-of-kin interview, indications of empty alcohol bottles used at the scene of death in combination with supplemental information included in the narrative, such as a toxicology report.	32.5%	29.9%	2.7 (−5.6, 10.9)	0.735
Acute substance use: The decedent was noted to have used at least 1 recreational/illicit drug the day before death or the day of death.	26.8%	23.9%	3.0 (−4.8, 10.7)	0.711
History of suicide attempt: The decedent was noted to have made at least 1 additional suicide attempt in the past, different from the attempt resulting in death.	10.0%	28.4%	−18.3 (−26.2, −10.5)	<0.001
Feeling depressed/melancholy: The decedent was noted to have been feeling depressed, dejected, down, or melancholy leading up to death (i.e., not a mental health/psychiatric condition).	28.6%	33.6%	−5.0 (−13.5, 3.4)	0.476
Psychiatric/mental health hospitalization: The decedent was noted to have had an inpatient hospitalization, rehabilitation, or residential treatment facility stay for psychiatric/mental health reasons at any time prior to death.	5.2%	9.7%	−4.5 (−9.7, 0.7)	0.093
Physical health				
General medical condition known: The decedent experienced a known physical health/general medical condition preceding death.	15.7%	16.4%	−0.7 (−7.3, 6.0)	0.868
Physical pain: The decedent was noted to have been experiencing physical pain preceding death.	5.2%	13.4%	−8.2 (−14.2, −2.3)	0.001
Sleep trouble: The decedent was noted to have experienced trouble sleeping, including conditions such as insomnia and sleep apnea, or sleeping more than they usually did.	—a	—a	—a	—a
Relationship dynamics				
Argument with romantic partner: The decedent was noted to have had an argument or dispute with their romantic partner (e.g., spouse, boyfriend, girlfriend, partner) during the days prior to death or day of death.	17.0%	23.9%	−6.9 (−14.5, 0.7)	0.120
Argument with nonromantic person: The decedent was noted to have an argument or dispute with someone other than a romantic partner (e.g., family member, friend, coworker) during the days prior to death or day of death.	3.4%	8.2%	−4.8 (−9.6, −0.1)	0.025
Romantic relationship challenges: The decedent was noted to have experienced problems in an established romantic relationship.	23.7%	24.6%	−0.9 (−8.6, 6.9)	0.868
Romantic relationship end/breakup: The decedent was noted to have experienced either the end of a romantic relationship or plans to end a romantic relationship preceding death.	18.3%	17.2%	1.2 (−5.6, 8.0)	0.865
Known/suspected infidelity: There were noted indications of either known or suspected infidelity, including romantic affairs and cheating.	5.3%	10.4%	−5.1 (−10.5, 0.2)	0.056
Final expressions of love: The decedent was noted to have expressed their love in combination with an expression of saying goodbye to family members, romantic partners, or friends within the days prior to death or the day of death.	—a	—a	—a	—a
Socioeconomic instability				
Job loss/unemployment: The decedent was noted to have experienced either a job loss or unemployment preceding death.	6.9%	9.0%	−2.1 (−7.2, 3.0)	0.600
Financial difficulties: The decedent was noted to have experienced a financial setback or financial distress preceding death.	—a	—a	—a	—a
Legal issues: The decedent was noted to have been experiencing legal troubles or distress preceding death, for example, being arrested, being accused of a crime, being sued, or having immigration-related legal issues.	10.2%	7.5%	2.8 (−2.0, 7.6)	0.567
Housing instability: The decedent was noted to have experienced homelessness or housing instability within the year prior to death.	—a	—a	—a	—a
Scene of death				
Found away from residence: The decedent was noted to have been found away from their primary residence, that is, public places, nature, other people's residences, and businesses.	25.3%	12.7%	12.6 (6.4, 18.8)	0.005
Found in automobile: The decedent was noted to have been found inside a car or other automobile either at or away from their primary residence.	12.5%	10.4%	2.0 (−3.6, 7.6)	0.732
Resuscitation attempt before death: There was a noted attempt to perform resuscitation at the time of death.	9.9%	9.7%	0.2 (−5.1, 5.6)	0.930
Firearm access and storage				
Another person’s firearm accessible: The decedent was noted to have accessed (i.e., borrowed or taken) the firearm involved in the fatal suicide attempt from a family member, romantic partner, friend, or nonfamily acquaintance. The firearm was not owned by the decedent.	4.6%	20.1%	−15.5 (−22.4, −8.6)	<0.001
Multiple firearms accessible: The decedent was noted to have multiple firearms accessible at the time of death.	—a	—a	—a	—a
Unsecure firearm storage: The decedent was noted to have not practiced secure firearm storage practices prior to death, including indications of keeping loaded firearms unlocked or stored in an open location in the home or vehicle.	2.0%	9.0%	−6.9 (−11.8, −2.0)	<0.001
Firearm acquired legally: The decedent was noted to have acquired the firearm used in the fatal suicide attempt following a legal process for purchasing a firearm.	—a	—a	—a	—a

*Note*: This table presents the authors' analysis of NVDRS data.

a Reflects data that are suppressed owing to cell count <10. There were 1,036 male decedents and 134 female decedents. The circumstances shown in this table are not mutually exclusive. The absence of a documented circumstance should not be interpreted as evidence that the circumstance did not occur but rather that it was not known or recorded in the available data. Risk differences (male minus female) with 95% CIs are shown; *p*-values were adjusted using the Benjamini–Hochberg FDR procedure. FDR, false discovery rate; NVDRS, National Violent Death Reporting System.

Male decedents in the sample were more commonly noted to have been found away from their residence than female decedents (25.3% vs 12.7%; RD=12.6, 95% CI=6.4, 18.8; FDR-adjusted *p*=0.005). In contrast, female decedents were more frequently noted to have accessed another person’s firearm (20.1% vs 4.6%; RD= −15.5, 95% CI= −22.4, −8.6; FDR-adjusted *p*<0.001) and to have indications of unsecured firearm storage (9.0% vs 2.0%; RD= −6.9, 95% CI= −11.8, −2.0; FDR-adjusted *p*<0.001). Indications of known or suspected infidelity were also more commonly noted among female than among male decedents in the sample; however, the CI included the null (RD= −5.1, 95% CI= −10.5, 0.2; FDR-adjusted *p*=0.056).

## DISCUSSION

This study inductively identified and descriptively characterized recurring circumstances preceding firearm suicide deaths among Hispanic/Latino adults in the U.S. Six major categories emerged to organize these circumstances: behavioral health, physical health, relationship dynamics, socioeconomic instability, scene of death, and firearm access and storage. Behavioral health circumstances were commonly noted among the decedents in the sample, including known mental health conditions and depressed or melancholy mood. Relationship and socioeconomic stressors were documented for a meaningful portion of the decedents in the sample, including romantic relationship challenges, interpersonal arguments, and legal issues. Notable sex differences were observed; female decedents in the sample were more frequently documented as having known mental health conditions, prior suicide attempts, and physical pain. Although descriptive, these findings provide population-specific context on circumstances preceding firearm suicide among Hispanic/Latino adults, a group historically underrepresented in the firearm suicide literature. This study also extends existing knowledge to a population for whom such evidence remains limited; many identified circumstances are consistent with prior research on suicide more broadly, underscoring their relevance within this population and helping to inform subsequent hypothesis-driven research. Additional research is warranted given recent firearm industry survey data indicating increases in firearm purchasing among Hispanic/Latino persons over the past 5 years.^6^

In addition to its substantive findings, this study also provides a methodologic contribution by demonstrating how qualitative content analysis of NVDRS narrative data can complement and contextualize the system’s existing variables. Specifically, many of the circumstances identified through this study’s coding procedures aligned closely with existing structured circumstance variables with similar definitions in the NVDRS. This provides assurance that the constructs derived from the narrative data in this study are consistent with the system’s established variable constructs and adds support to the overall quality of the NVDRS abstraction process. At the same time, the narrative data allow researchers to potentially identify variations in language, level of detail, and content across decedents that are not reflected in the system’s structured variables. This may be relevant for research involving racial/ethnic minority decedents, given documented differences across racial/ethnic groups in narrative length, lexical diversity, and descriptive detail.^31^^,^^32^ Overall, this study’s findings highlight both the utility of NVDRS narrative data and the value of integrating qualitative approaches to enhance the interpretation of the existing surveillance variables. Future work could build on this approach by leveraging manually coded data to train and validate natural language processing approaches for identifying recurring circumstances in NVDRS narrative data.

Previous research has shown that firearm owners are more likely than nonowners to be impulsive, which is related to the capacity for suicide.^40^, ^41^, ^42^ In this study, legal issues (∼10%) and arguments with romantic partners (∼18%) were repeatedly documented among the suicide decedents in the sample. As such, this study may have included individuals who attempted suicide with firearms not because of mental ill health but because they acted impulsively during acute emotional crises that felt overwhelming in the moment. For example, prior research suggests that immigration-related legal distress may adversely affect mental health and suicidal ideation^13^ and, when present, may constitute an acute emotional crisis preceding death among some Hispanic/Latino adults. In this study, immigration-related concerns were included within the broader legal issues circumstance for a subset of decedents and were not the most common legal stressor; thus, this observation should be viewed as hypothesis generating. However, it warrants further investigation into the types of immigration- and nonimmigration-related legal issues experienced by Hispanic/Latino adults who die by firearm suicide.

Furthermore, acute alcohol (∼32%) and substance use (∼27%) were repeatedly noted prior to death in the study sample, which is consistent with findings from more general firearm suicide studies.^43^^,^^44^ The use of such substances—potential disinhibitors—may facilitate impulsive suicide attempts during acute emotional crises. However, the relationships among substance use, impulsivity, and suicidality are complicated,^45^, ^46^, ^47^ and it is important to reiterate that acute substance use was identified in this study from narrative documentation and may not reflect substance quantity or blood alcohol concentration at the time of death.

Compared with previous studies, this study’s results suggest that known mental health conditions may be less common among Hispanic/Latino firearm suicide decedents (i.e., 26.4% of decedents) than among suicide decedents in general (about 35%–50%),^48^ although mental health condition definitions vary across previous studies. Notably, the lower prevalence of documented mental health conditions among firearm suicide decedents than among nonfirearm suicide decedents has been observed in other populations and is therefore not unique to Hispanic/Latino persons.^44^^,^^49^, ^50^, ^51^, ^52^ This pattern should also be interpreted with caution: prior research indicates that many individuals who die by suicide have limited or no contact with mental health care before death,^53^ which may contribute to the underdiagnosis or underdocumentation of mental health conditions in surveillance data. Accordingly, the lower prevalence observed in this study may reflect differences in detection, diagnosis, or health-seeking behavior rather than the absence of underlying mental health needs.

Relatedly, this study found that less than one third of Hispanic/Latino firearm suicide decedents in the sample were noted to have been feeling depressed or melancholy in the period preceding death, irrespective of known psychiatric conditions. Although not all individuals who experience periods of depressed mood or emotional distress meet the criteria for a psychiatric diagnosis, this finding may also indicate that individuals in the data may have experienced undiagnosed mental health conditions or been unable to access needed mental health services. Such insights seem to be consistent with the literature on living Hispanic/Latino persons: previous studies have demonstrated that Hispanic persons are less likely than non-Hispanic persons to access mental health care,^54^ even when psychiatric illness is present, owing to cultural stigma, limited healthcare options, legal concerns, and language or literacy barriers.^55^, ^56^, ^57^, ^58^

A low proportion of Hispanic/Latino firearm suicide decedents in this study had a documented history of previous suicide attempts, and an even lower proportion were noted to have been previously hospitalized for mental health reasons. Although previous studies have shown that suicide decedents who die by firearm are less likely to have made a previous attempt than those who die by other methods,^42^^,^^51^^,^^59^ this study’s finding on the commonality of documented psychiatric hospitalization prior to firearm suicide death appears to be distinctive. It is well established that there is an elevated suicide risk among persons with previous suicide attempts^60^ and in the 30 days after psychiatric discharge.^61^ However, this study’s findings suggest that firearm suicide deaths among Hispanic/Latino adults may occur without these traditional risk factors. Moreover, these findings likely indicate challenges for prevention, because many Hispanic/Latino persons who die by firearm suicide may never access mental health services, even when they may have benefited from hospitalization or other services during periods of acute distress. Consequently, such individuals may be missed by conventional suicide prevention protocols that rely on specific diagnoses, documented crises, or postdischarge follow-up, which likely underscores a need for community-based prevention efforts delivered in culturally familiar and trusted community settings.

Of relevance to future studies focusing on prevention, lethal means assessment and counseling (LMC) is a promising strategy that can help clinicians work with patients to improve firearm safety in the home or voluntarily limit firearm access before suicidal crises emerge. Although LMC has been shown to improve safe firearm storage practices^62^ and may reduce the risk of suicide attempts,^63^ its implementation often depends on detecting traditional warning signs, such as mental illness, substance use, or suicidal ideation. Although only descriptive, this study’s findings indicate that additional circumstances, including relationship conflict or legal stressors, were documented among Hispanic/Latino firearm suicide decedents and may warrant consideration when assessing opportunities for LMC among Hispanic/Latino adults. In addition to healthcare settings, culturally responsive outreach in community contexts where Hispanic/Latino community members often seek wellness-related support (e.g., botánicas, Ventanilla de Salud program administered through Mexican Consulates^64^^,^^65^) may expand the reach of LMC and safe firearm storage initiatives. However, these observations should be considered hypothesis generating, warranting further investigation. Broadly, these findings support the importance of future work to develop and test culturally and contextually tailored firearm suicide prevention strategies in Hispanic/Latino communities.^66^

Finally, several findings differed by sex. Known mental health/psychiatric conditions and prior suicide attempts were more commonly noted among the female decedents than among the males included in the study sample. Consistent with previous studies, these findings may suggest that mental health stigma is particularly salient—or that mental healthcare accessibility is particularly challenging—among Hispanic/Latino males at risk of firearm suicide death relative to Hispanic/Latina females.^67^ These findings may also suggest that Hispanic/Latino males who die by firearm suicide are more likely than Hispanic/Latina females to access and use firearms (i.e., the most lethal suicide method^68^) during their initial suicide attempts. Notably, female decedents in the study sample were more frequently noted to have accessed another person’s firearm and to have indications of unsecured firearm storage. These findings appear to be consistent with recent research demonstrating that secondary exposure to household firearms is associated with higher suicide rates among women in California who do not personally own handguns^21^—and thus suggest that secondary exposure to household firearms warrants further investigation as a potential target for suicide prevention among Hispanic/Latina women. In contrast, male decedents were more frequently noted to have been discovered away from their residence, a pattern that may reflect different situational or behavioral circumstances preceding death and that warrants additional research.

### Limitations

This study had several strengths. First, it used population-based data from the NVDRS, a unique state-based violent death surveillance system that links information from vital records and investigative reports to provide comprehensive incident-level data. Second, the qualitative content analysis of CME/LE narrative text data used a systematic inductive code development process followed by deductive application with high interrater reliability, allowing for rigorous and reproducible characterization of circumstances that recurred prior to firearm suicide death among a large sample of Hispanic/Latino adults. By grounding circumstance definitions directly in CME/LE narrative text data rather than relying solely on predefined structured variables, the analysis centered the documented language and context of Hispanic/Latino firearm suicide decedents and allowed for careful assessment of contextual nuance and population-specific framing. Notably, the study findings aligned with existing NVDRS variables, providing insight into the quality and utility of NVDRS data. Third, the probability sampling approach permitted analysis of a large corpus of narrative data while appearing to maintain representativeness of Hispanic/Latino adult firearm suicide decedents in NVDRS from 2013 to 2021 on measured characteristics, although statistical generalization was not the primary objective of the qualitative analysis. Fourth, integrating qualitative coding with quantification enabled descriptive characterization of circumstances and sex differences in a manner that can inform subsequent hypothesis-driven analyses. Finally, the systematic manual review of a large corpus of CME/LE narrative text further strengthened the depth and interpretability of the findings.

This study had several limitations. First, there is inherent variability in the CME/LE incident narratives, which may reflect differences in medicolegal death investigation practices across jurisdictions. Prior research has documented differences in the presence, length, and informational content of NVDRS narratives by racial identity,^31^^,^^32^ and a recent national evaluation from the National Academies of Sciences, Engineering, and Medicine describes the fragmentation and inconsistencies in the U.S. medicolegal death investigation system.^69^ Consequently, variability stemming from the fragmented medicolegal death investigation process may have led to underascertainment or differential descriptions of some circumstances in the narrative text. The study findings should be interpreted with caution.

Second, it is important to note that this study’s results are descriptive (i.e., not causal) and do not measure suicide risk. This study examined decedent data and should not be used to predict suicidal behavior in living populations. Moreover, this study only sought to describe the Hispanic/Latino firearm suicide decedents represented in the study sample, contributing to this nascent literature. This study did not include a nonfirearm comparison group because the objective was to characterize circumstances within firearm suicide decedents rather than to compare across mechanisms of death. Understanding the differences in the distributions of characteristics between Hispanic/Latino adult suicide decedents and living Hispanic/Latino adults would better inform suicide prevention opportunities. More research is necessary before firm conclusions about suicide prevention within this population are drawn.

Third, statistical generalization was not the primary objective of this qualitative analysis; therefore, the study findings should be interpreted with caution and not be extrapolated to all Hispanic/Latino adults. Nevertheless, the probability sampling approach and representativeness checks support the broader descriptive relevance of the findings. Notably, many of the circumstances identified through the coding procedures aligned with existing NVDRS structured circumstance variables with similar definitions for decedents in the sample (e.g., financial problems [7.0%], housing instability [2.6%], history of suicide attempt [13.1%], and known mental health problems [31.3%]). This alignment provides reassurance that this study’s approach captured constructs consistent with established NVDRS circumstance variables, while allowing for greater contextual specificity and alternative characterizations of these circumstances, although some individual experiences may remain underrepresented in the narrative data.

Fourth, suicide is a complex and multifaceted phenomenon influenced by often co-occurring biopsychological and socioeconomic factors. Although this study presents each circumstance as a discrete phenomenon, the circumstances are not mutually exclusive. Later phases of this program of research will assess the relationships among these circumstances and other factors to better inform lessons for suicide prevention among Hispanic/Latino adults.

Fifth, the NVDRS program synthesizes the narrative data from CME/LE investigative reports, incorporating information primarily derived from interviews with the decedents’ next of kin. This process may cause several issues with what information was reported through the CME/LE narratives. Next-of-kin interviews may be subject to recall bias, or different types of informants may provide more or less salient circumstance information depending on their relationship and proximity to the decedent. Thus, it is possible that the decedents’ next of kin may have been unaware of specific but salient circumstances experienced by the decedents before death. Notably, NVDRS abstractors commonly have the opportunity to incorporate information from other documents (e.g., toxicology and medical data) into the CME/LE narratives, whether included with or in addition to the investigation reports. Still, the absence of a circumstance in the narrative may not mean that it was not present for the decedent; it is possible that certain information may have eluded the CME/LE investigation or NVDRS abstraction process.

Sixth, suicide death or ethnicity may be underreported owing to limited information, the attentiveness of CMEs, or the misclassification of the cause of death or ethnicity on Hispanic/Latino adults’ death records. To help address these concerns, the NVDRS program uses multiple data sources to improve demographic classification. However, it is possible that Hispanic/Latino suicide decedents may have been missing from the data before they were received; the circumstances experienced by such decedents may have differed from those experienced by the decedents included in this study.

## CONCLUSIONS

This analysis of Hispanic/Latino adult firearm suicide decedents in the NVDRS describes recurring health, relationship, socioeconomic, and firearm access circumstances, several of which vary in commonality by sex. Notably, traditional clinical indicators, such as prior psychiatric hospitalization or known mental health diagnoses, were not documented for many decedents in the study sample, and female decedents were more likely to have been noted to have accessed another person’s firearm. This study’s findings highlight the need for further investigation into the circumstances and potential risk factors experienced by this population and for additional evaluation of when interventions, such as LMC, should be implemented. This study also demonstrates the value of leveraging NVDRS narrative data to complement structured variables and offers insight into the quality and utility of the NVDRS abstraction process and data.

## CRediT authorship contribution statement

**Alexys M. Abraham:** Formal analysis, Investigation, Software, Data curation, Validation, Writing – original draft, Writing – review & editing. **Elise V. Bailey:** Writing – original draft. **Aryanna Sanger:** Formal analysis, Software, Validation. **Diana E. Naranjo:** Conceptualization, Formal analysis, Writing – review & editing. **Fernando A. Wilson:** Conceptualization, Writing – review & editing. **Evan V. Goldstein:** Conceptualization, Data curation, Formal analysis, Funding acquisition, Investigation, Methodology, Project administration, Resources, Supervision, Validation, Visualization, Writing – original draft, Writing – review & editing.

## Declaration of interest

EVG reports that financial support was provided by NIH. EVG reports a relationship with Kaiser Permanente Center for Gun Violence Research & Education that includes funding grants; he received a grant from this sponsor. No other financial disclosures were reported.

## Appendix Supplementary materials

Image, application 1
