# Supplementary material for: Circumstances Preceding Firearm Suicide Deaths in a Large Sample of Hispanic/Latino Adults: Findings From a Content Analysis
Source: AJPM Focus. 2026 Jul 15;5(6):100531. doi: 10.1016/j.focus.2026.100531 (PMC13634510; doi:10.1016/j.focus.2026.100531)
Supplement: Supplementary file 1 [file mmc1.pdf]

ARTICLE TITLE: Circumstances Preceding Firearm Suicide Deaths in a Large Sample of Hispanic/Latino Adults: Findings from a Content Analysis
AUTHORS: Abraham A, Bailey EV, Sanger A, Naranjo DE, Wilson FA, Goldstein EV
JOURNAL: AJPM Focus

APPENDIX

Addition Details: Content Analysis Procedures & Research Team Positionality

The study team conducted qualitative content analysis with quantification[1–3] to identify, describe, and enumerate the recurring circumstances the decedents experienced prior to death, as summarized through the CME/LE narratives. The CME/LE narrative was the unit of analysis. This process involved three systematic phases. For the first phase, the study team did not use a predetermined list of circumstances preceding death to code the incident narratives. Instead, two members of the research team independently reviewed a random sub-sample of 100 decedents and inductively coded all emergent, recurring circumstances preceding death as described in each decedent's CME/LE narrative. The researchers then compared, discussed, reconciled, named, and defined the most commonly occurring circumstance codes. The researchers created a first-cycle codebook with clear definitions for each code.[4]

During this part of the process, the researchers analyzing the data shared their positionality, wrote reflexive memos, and met weekly to document and share their reactions to the data and methods.[5,6] The purpose of these reflexive activities was to acknowledge the researchers' personal thoughts and reactions during data analysis, reflect on potential biases, and limit their positionality from influencing how they interpreted meaning from the subjective truths experienced by the participants. Hispanic/Latino/a voices – both male and female – were represented in the analysis and writing of this manuscript, potentially connecting to the cultural identities of those persons represented in the analytic samples. The professional identities of the

study team members include backgrounds in social science, criminology, social work, and public health. The researchers identify with a range of theoretical philosophies, including constructivism, empiricism, pragmatism, justice. Although this study was not guided by critical theory, nor was there any interaction with human subjects, the researchers took an equity-centered approach to the analysis, remaining highly attentive to the potential power relations inherent in this work. As best as possible, the study team described the experiences of the decedents represented in the study without extrapolating beyond the details included in the CME/LE narratives or making comparisons to other populations.

For the second phase, two members of the research team used the codebook to deductively apply the existing codes to the full analytic sample. To ensure the reliability of the deductive coding process, the study team members analyzing the data held weekly meetings to review ongoing code applications and discuss and reconcile all cases for which the applicability of any code was uncertain. The potential emergence of any new, salient codes was also discussed and tracked weekly, as were any necessary refinements to the code definitions. Any code definition refinements prompted an immediate review of all previous cases for the code across both analytic samples. All coding was conducted at a semantic level[7,8] to stay as true as possible to the decedents' documented experiences without extrapolating beyond the NVDRS abstractors' reporting of each death. Given the probability sampling approach and sample sizes, and assuming the probability of the rarest relevant circumstances occurring was between 1 in 100 and 1 in 1,000 cases, the researchers expected >95% certainty that the samples included at least one of the rarest relevant circumstances.[3] Notably, the absence of a documented circumstance should not be interpreted as evidence that the circumstance did not occur, but rather that it was not known or recorded in the available data.

As final reliability checks, random case selections were checked for coding accuracy after all cases were coded. Additionally, interrater reliability was assessed across the applied codes for a random sample of 100 incident narratives per analytic sample. The average percent agreement between coders was 95.7% for the firearm suicide decedent selections, indicating a high level of consistency in the deductive coding decisions. To account for potential chance agreement – especially given the imbalanced prevalence of some codes – the study team also calculated Gwet's AC1 across all codes. The resulting AC1 values of 0.77 for the firearm suicide decedent selections, reflects substantial overall agreement between coders and provides a more robust estimate of reliability than raw agreement alone. These tests gave us further confidence that the application of the agreed-upon coding scheme was clear and consistent.

References

1. Christensen BM, Bjällmark A, Ndipen IM, Afram SS, Bazzi M. Barriers to radiographers' use of radiation safety principles: A qualitative perspective. *J Med Radiat Sci*. 2024;71(2). doi:10.1002/jmrs.750
2. Lederer AM, Sheena BS. A content analysis of college students' gaps in knowledge about sexually transmitted infections. *Health Educ J*. 2021;80(2). doi:10.1177/0017896920959091
3. Krippendorff K. *Content Analysis : An Introduction to Its Methodology*. 2nd ed. Sage; 2004.
4. Saldaña J. *The Coding Manual for Qualitative Researchers*. 3rd ed. SAGE Publications Inc; 2016.
5. Guillemin M, Gillam L. Ethics, reflexivity, and "Ethically important moments" in research. *Qualitative Inquiry*. 2004;10(2):261-280. doi:10.1177/1077800403262360
6. St. Louis K, Barton AC. Tales from the science education crypt: A critical reflection of positionality, subjectivity, and reflexivity in research. *Forum Qualitative Sozialforschung*. 2002;3(3).
7. Miles MB, Huberman AM. *Qualitative Data Analysis Second Edition. SAGE Publications.* 1994.
8. Creswell JohnW, Poth CN. *Qualitative Inquiry & Research Design: Choosing among 5 Approaches*. 4th ed. SAGE Publications Inc.; 2018.

Table A1. Comparison of Measured Characteristics Between Analytic Sample and Non-Sampled Hispanic Adult Firearm Suicide Decedents, 2013–2021

	Sample (n = 1,170)		Not-in-Sample (n = 4,970)			
	Mean/ Frequency	Standard Deviation/ Percent	Mean/ Frequency	Standard Deviation/ Percent	SMD	ASD
Age in years, mean, SD	38.4	16.5	38.1	16.5	0.017	0.017
Sex, n, %						
Male	1,036	88.5	4,420	88.9	-0.012	0.012
Female	134	11.5	550	11.1	0.012	0.012
Education Level, n, %						
8th grade or less	101	8.6	349	7.0	0.060	0.060
9th to 12th grade, no diploma	171	14.6	738	14.8	-0.007	0.007
High school graduate or GED completed	460	39.3	2,047	41.2	-0.038	0.038
Some college credit, but no degree	209	17.9	887	17.8	0.000	0.000
Associate's degree	67	5.7	334	6.7	-0.041	0.041
Bachelor's degree	80	6.8	284	5.7	0.046	0.046
Master's degree	13	1.1	91	1.8	-0.060	0.060
Doctorate or Professional degree	10	0.9	29	0.6	0.032	0.032
Unknown	43	3.7	179	3.6	0.004	0.004
Marital Status, n, %						
Married/Civil Union/Domestic Partnership	348	29.7	1,485	29.9	-0.003	0.003
Never Married	572	48.9	2,433	49.0	-0.001	0.001
Widowed	37	3.2	114	2.3	0.053	0.053
Divorced	148	12.6	672	13.5	-0.026	0.026

Married/Civil Union/Domestic Partnership, but separated	35	3.0	140	2.8	0.010	0.010
Single, not otherwise specified	19	1.6	71	1.4	0.016	0.016
Unknown	11	0.9	54	1.1	-0.015	0.015
Military Veteran Status, n, %						
Did not serve	979	83.7	4,151	83.5	0.004	0.004
Served in the US Armed Forces	170	14.5	727	14.6	-0.003	0.003
Unknown	20	1.7	86	1.7	-0.002	0.002
Death Year, n, %						
2013	39	3.3	179	3.6	-0.015	0.015
2014	60	5.1	190	3.8	0.063	0.063
2015	79	6.8	270	5.4	0.055	0.055
2016	94	8.0	401	8.1	-0.001	0.001
2017	126	10.8	531	10.7	0.003	0.003
2018	164	14.0	670	13.5	0.016	0.016
2019	145	12.4	641	12.9	-0.015	0.015
2020	214	18.3	940	18.9	-0.016	0.016
2021	249	21.3	1,148	23.1	-0.044	0.044
Region, n, %						
Mountain	474	40.5	1,861	37.4	0.063	0.063
East North Central	125	10.7	432	8.7	0.067	0.067
East South Central	17	1.5	84	1.7	-0.019	0.019
Middle Atlantic	64	5.5	261	5.3	0.010	0.010
New England	28	2.4	98	2.0	0.029	0.029
Pacific	171	14.6	798	16.1	-0.040	0.040
South Atlantic	115	9.8	557	11.2	-0.045	0.045
West North Central	47	4.0	202	4.1	-0.002	0.002
West South Central	96	8.2	540	10.9	-0.091	0.091
Puerto Rico	33	2.8	137	2.8	0.004	0.004

Notes: Authors' analysis of NVDRS data. Analytic sample compared with all other Hispanic adult firearm suicide decedents in NVDRS (2013–2021), including decedents with missing CME/LE narrative data. Absolute standardized differences (ASDs) were used to compare sampled and non-sampled individuals; values <0.10 indicate negligible imbalance.

Table A2. States and Contributing Years of Firearm Suicide Decedents in the Analytic Sample

State	Range (Years)
Alabama	2018-2019
Alaska	2014, 2016-2017, 2019, 2021
Arizona	2015-2021
California	2017-2021
Colorado	2013-2021
Connecticut	2015-2016, 2018-2019, 2021
District of Columbia	2019, 2021
Georgia	2013-2021
Hawaii	2015, 2019
Idaho	2020-2021
Illinois	2016-2021
Indiana	2016-2021
Iowa	2018, 2020-2021
Kansas	2015, 2017-2021
Kentucky	2014-2015, 2020-2021
Louisiana	2018, 2020-2021
Maine	2021
Maryland	2014, 2017, 2020-2021
Massachusetts	2013-2015, 2017-2021
Michigan	2014-2021
Minnesota	2015-2018, 2021
Missouri	2019-2021
Montana	2020-2021
Nebraska	2018, 2020
Nevada	2017-2021
New Jersey	2013-2014, 2016-2021

New Mexico	2013-2021
New York	2015-2018, 2020-2021
North Carolina	2013-2021
North Dakota	2021
Ohio	2013, 2015-2021
Oklahoma	2013-2016, 2018-2021
Oregon	2014-2021
Pennsylvania	2016-2017, 2019-2021
Rhode Island	2018, 2021
South Carolina	2013-2014, 2016-2019
South Dakota	2020
Tennessee	2020-2021
Texas	2020-2021
Utah	2013, 2015-2021
Virginia	2013-2021
Washington	2016-2021
West Virginia	2017
Wisconsin	2014-2021
Wyoming	2020
Puerto Rico	2017-2021

Table A3. Code definitions/criteria

CODE	DEFINITION
acute alcohol use	The decedent was noted to have used alcohol the day of death. This may be indicated by a next-of-kin interview, indications of empty alcohol bottles used at the scene of death in combination with supplemental information included in the narrative, such as a toxicology report.
argument with non-romantic partner	The decedent was noted to have an argument or dispute with someone other than a romantic partner (e.g., family member, friend, coworker) during the days prior to death or the day of death.
argument with romantic partner	The decedent was noted to have an argument or dispute with their romantic partner (e.g., spouse, boyfriend, girlfriend, partner) during the days prior to death or the day of death. Note: This alone is NOT necessarily also an indication of a "romantic relationship issues."
acute substance use	The decedent was noted to have used at least one recreational/illicit drug the day before death or the day of death, such as, but not limited to, THC, cocaine, methamphetamine, heroin, MDMA. This does NOT include prescription medications/drugs. This can be indicated by a next-of-kin interview or supplemental information included in the narrative, such as a toxicology report.
substance/alcohol use history	The decedent was noted to have experienced a known substance use disorder (SUD), alcoholism, *or* past/patterned/recent history of drug or alcohol use, either indicated by next-of-kin or supplemental information included in the narrative (e.g., medical history/ information). This is NOT alcohol or drug use during the day of the fatal suicide attempt (see "alcohol use" and "drug use").
Final expression of love	The decedent was noted to have expressed their love in combination with an expression of saying goodbye to family members, romantic partners, or friends within the days prior to death or the day of death. The expression of love may have been conducted in person, over the phone, via text message, or through social media.
feeling depressed/melancholy	The decedent was noted to have been feeling depressed, dejected, morose, despondent, down, melancholy, etc., during the day of death or in the weeks and/or days leading up to death. This does NOT include indications of known mental health/ psychiatric conditions or disorders (see "mental health condition known"), but both codes can be applied at the same time.
financial difficulties	The decedent was noted to have experienced a financial/economic setback or financial/economic distress within the months, weeks, and/or days prior to death. This does NOT include job or employment-related difficulties (see "job difficulties/stress" and "job loss/unemployment").

found away from residence	The decedent was noted to have been found away from their primary residence, i.e., public places, nature, other people's residences, and businesses.
found in automobile	The decedent was noted to have been found inside a car or other automobile either at or away from their primary residence. Note: If the decedent’s car was located on site at the decedent’s residence, then the "found at residence" code may also be applied. If the decedent’s car was located away from the decedent’s residence, then the "found outside of residence" code may also be applied.
general medical condition known	The decedent experienced a known physical health/general medical condition within the year leading up to death, as indicated by next-of-kin or supplemental information included in the narrative (e.g., medical history/information). E.g., chronic illnesses and diseases like hypertension and diabetes mellitus, kidney disease/renal failure, cancer, heart disease, obesity, liver disease, arthritis, asthma. This does NOT include mental health or psychiatric conditions. This may be indicated by (1) the mentioning of a specific diagnosis, (2) receiving treatment for physical health/general medical issues, (3) medical history included in the narrative document, (4) individuals in the narrative documents indicating a decedent experienced physical health/general medical issues and received care for those issues leading up to death. Do NOT code if it was explicitly stated that the condition occurred >1 year prior; however, yes code if no specific timeframe was given and it was noted that the condition happened recently, lately, etc., or was ongoing.
history of suicide attempt	The decedent was noted to have made at least one additional suicide attempt in the past, different from the attempt resulting in death. This does NOT include the most recent suicide attempt that resulted in death.
housing instability	The decedent was noted to have experienced homelessness or housing instability within the year prior to death. Housing instability includes the known or expected loss of an apartment, house, or other home, which may be indicated by eviction, foreclosure, or the inability to pay mortgage or rent. Housing instability may also be indicated by the necessity of living with family members or friends due to high housing costs. Note: Homelessness may be referred to as “being transient.”
job loss/unemployment	The decedent was noted to have experienced either (1) job loss or (2) unemployment within the months, weeks, and/or days prior to death. This does NOT include other difficulties or stresses in the workplace (see "job difficulties/stress").
known/suspected infidelity	There were noted indications of either (1) known or (2) suspected infidelity, including romantic affairs and cheating within the year prior to death. This includes known/suspected infidelity committed by either the decedent OR the decedent's romantic partner. This includes marriages and all other romantic relationships. Note: If this specific code is applied, then the "romantic relationship issues" code should also be applied.

legal issues	The decedent was noted to have been experiencing legal troubles or distress in the months, weeks, and/or days prior to death. This may include, for example, court hearings, being accused of a crime, being sued, fraud, sexual misconduct allegations, etc. This does NOT include indications of known, past criminal history (see "criminal history") or actively committing a crime (see "committing crime").
mental health condition known	The decedent was noted to have experienced a mental health/psychiatric condition or disorder within the year leading up to death, as indicated by next-of-kin or supplemental information included in the narrative (e.g., medical history/information). This may be indicated by (1) the mentioning of a specific diagnosis, (2) receiving treatment for mental health issues, (3) medical history included in the narrative document, (4) individuals in the narrative documents indicating a decedent experienced mental health/psychiatric issues and received care for those issues leading up to death. This does NOT include only indications of decedents feeling depressed, melancholy, or down (see "feeling depressed/melancholy/ stressed"), or sleep or eating disorders. Do NOT code if it was explicitly stated that the condition occurred >1 year prior; however, yes code if no specific timeframe was given and it was noted that the condition happened recently, lately, was ongoing, or had a history of the mental health condition (i.e., “history of depression”). Examples of mental health conditions include (but are not limited to) mood disorders (depression, major depressive disorder, bipolar disorder, persistent depressive disorder), anxiety disorders (generalized anxiety disorder, panic disorder, post-traumatic stress disorder [PTSD], psychotic disorders (schizophrenia, delusional disorder), obsessive compulsive disorder (OCD), delusional disorder, dissociative disorders, and eating disorders.
physical pain	The decedent was noted to have been experiencing physical pain within the months, weeks, and/or days preceding death. Note: This code can be applied together with "general medical condition known (non-mental health)" or "mental health condition known."
psychiatric/mental health hospitalization	The decedent was noted to have had an inpatient hospitalization, rehabilitation, or residential treatment facility stay for psychiatric/mental health reasons at any time prior to death. Note: Terminology such as “placing a person/patient on a hold” and “voluntary or involuntary hold” may indicate a mental health hospitalization or treatment stay, as jurisdiction-specific terminology such as “blue sheet, “pink slip,” “5150,” or “Baker Act,” depending on the state.
resuscitation attempt before death	There was a noted attempt to perform resuscitation before the decedent died. This may include CPR or other acts of reviving someone from a life-threatening situation. This can include resuscitations efforts performed by Emergency Medical Services (EMS) personnel, such as transporting the decedent to a medical facility via ambulance prior to dying, or family, friends, coworkers, romantic partners, and other people.
romantic relationship end/breakup	The decedent was noted to have experienced either (1) the end of a romantic relationship or (2) plans to end a romantic relationship. This includes marriages and all other romantic relationships. This includes indications of, for example, recent or planned separation, divorce, annulment, and break up within the year prior to death. Note: If this specific code is applied, then the "romantic relationship issues" code should also be applied.

romantic relationship challenges	The decedent was noted to have experienced problems in an established romantic relationship, situationship, or marriage within the months, weeks, and/or days prior to death. This does NOT include ONLY a single indication of an interpersonal argument (see "argument with romantic partner"). Rather, this includes indications of a deeper-rooted/patterned trouble in the relationship, situationship, or marriage or indications that the problem could threaten the continuation of the relationship. E.g., indications of known or suspected infidelity, separation, break up, or divorce.
sleep trouble	The decedent was noted to have experienced trouble sleeping, including conditions like insomnia and sleep apnea, or sleeping more than they usually did. This can be indicated by next-of-kin or supplemental information included in the narrative (e.g., medical history/information).
another person's firearm accessible	The decedent was noted to have accessed (i.e., borrowed or taken) the firearm involved in the fatal suicide attempt from a family member, romantic partner, friend, or non-family acquaintance. The firearm was not owned by the decedent. Note: This includes indications that the firearm was stolen, taken, or borrowed from someone else, or otherwise accessible to the decedent prior to death.
multiple firearms accessible	The decedent was noted to have multiple firearms accessible at the time of death.
unsecure firearm storage	The decedent was noted to have not practiced secure firearm storage practices prior to death, including indications of keeping loaded firearms unlocked or stored in an open location in the home or vehicle.
firearm acquired legally	The decedent was noted to have acquired the firearm used in the fatal suicide attempt following a legal process for purchasing a firearm.
